# Supplementary material for: Transcriptomic Analysis Provides Novel Insights into Heat Stress Responses in Sheep
Source: Animals (Basel). 2019 Jun 24;9(6):387. doi: 10.3390/ani9060387 (PMC6617286; doi:10.3390/ani9060387)
Supplement: Supplementary file 1 [file animals-09-00387-s001.zip › Supplementary/Supplementary Table S1.docx]

**Table S1** **Primers used in this study for qRT-PCR.**

| Gene | Primer sequences (5'-3') | Accession No. | Products length (bp) |
| --- | --- | --- | --- |
| *HTR4* | ATAGGAACAAGATGACCCCTC | XM_015096127.1 | 106 |
|  | ATGTTATTCCAGCCTTGCATT |  |  |
| *SLC13A5* | GATCTTGTTCCTCACCCCGAT | XM_004012581.3 | 103 |
|  | CCAGTAAACGGCCATGACGAT |  |  |
| *GPX3* | GCTGGCAAATACATCCTCTTCGT | XM_015096153.1 | 104 |
|  | AGACCGAATGGTTCAAGCTCT |  |  |
| *NPFFR2* | CCTTTTGGAAGTACAATGTGC | KC119399.1 | 160 |
|  | TAATGATGACAAATGCCGTCT |  |  |
| *NPR1* | ATTATGGCTCCCTACTGACCAC | XM_015092256.1 | 105 |
|  | TGCGTTTCCGGTTCACACGTT |  |  |
| *CPVL* | AGGTTTCAGTTTTACCGACCA | XM_015095331.1 | 156 |
|  | TACTTCCCAGCGTAAGACTCC |  |  |
| *UPP2* | TGGCTTCGATTTTACCTGCT | XM_012136549.2 | 168 |
|  | CAAACATTTCCGGTAGGTTG |  |  |
| *HTR1B* | GCCAACTACCTGATCGCCTCC | XM_015097299.1 | 129 |
|  | CAGCCAGAAGTCGCAGACCAC |  |  |
| *FMO5* | TGTGCTTCAGTGACTATCCCA | XM_012179729.2 | 200 |
|  | CCCTTCAGATTCCGTGACCA |  |  |
| *VIL1* | ACAAGACCTGCTCAGTCACGA | XM_004004924.2 | 183 |
|  | GCCCCGTCATTCTGAAGCTC |  |  |
| *ACTB* | CCAACCGTGAGAAGATGACC | U39357.2 | 97 |
|  | CCCGAGGCGTACAGGGACAG |  |  |

*HTR4*: 5-Hydroxytryptamine receptor 4; *SLC13A5*: Sodium-dependent citrate transporter member 5; *GPX3*: Glutathione peroxidase 3; *NPFFR2*: Neuropeptide FF receptor 2; *NPR1*: Natriuretic peptide receptor 1; *CPVL*: Carboxypeptidase vitellogenic-like; *UPP2*: Uridine phosphorylase 2; *HTR1B*: 5-Hydroxytryptamine receptor 1B; *FMO5*: Flavin-containing monooxygenase 5; *VIL1*: Villin 1.
