# Supplementary material for: Transcriptomic Analysis Provides Novel Insights into Heat Stress Responses in Sheep
Source: Animals (Basel). 2019 Jun 24;9(6):387. doi: 10.3390/ani9060387 (PMC6617286; doi:10.3390/ani9060387)
Supplement: Supplementary file 1 [file animals-09-00387-s001.zip › Supplementary/Supplementary Table S2.docx]

**Table S2 Number of genes in different FPKM intervals.**

| FPKM  interval | CG1 | CG2 | CM1 | CM2 | HG1 | HG2 | HM1 | HM2 |
| --- | --- | --- | --- | --- | --- | --- | --- | --- |
| 0.1~1 | 9,462  (48.09%) | 10,721 (54.49%) | 9,281 (47.17%) | 9,181 (46.66%) | 8,891 (45.19%) | 9,051 (46%) | 9,099 (46.25%) | 8,886  (45.16%) |
| 1~5 | 4,291 (21.81%) | 3,835 (19.49%) | 4,809 (24.44%) | 4,665 (23.71%) | 4,917 (24.99%) | 4,723 (24.01%) | 4,709 (23.93%) | 4,834 (24.57%) |
| 5~10 | 1,818  (9.24%) | 1,504  (7.64%) | 1,874  (9.52%) | 1,926  (9.79%) | 2,018 (10.26%) | 1,915  (9.73%) | 1,918  (9.75%) | 1,971  (10.02%) |
| 10~20 | 1,465  (7.45%) | 1,207  (6.13%) | 1,401  (7.12%) | 1,493  (7.59%) | 1,529  (7.77%) | 1,513  (7.69%) | 1,490  (7.57%) | 1,474  (7.49%) |
| 20~30 | 657  (3.34%) | 567  (2.88%) | 600  (3.05%) | 612  (3.11%) | 616  (3.13%) | 660  (3.35%) | 611  (3.11%) | 656  (3.33%) |
| 30~40 | 351  (1.78%) | 309  (1.57%) | 333  (1.69%) | 371  (1.89%) | 348  (1.77%) | 383  (1.95%) | 362  (1.84%) | 363  (1.84%) |
| 40~50 | 247  (1.26%) | 205  (1.04%) | 205  (1.04%) | 221  (1.12%) | 212  (1.08%) | 218  (1.11%) | 220  (1.12%) | 232  (1.18%) |
| >50 | 1,384  (7.03%) | 1,327  (6.74%) | 1,172  (5.96%) | 1,206  (6.13%) | 1,144  (5.81%) | 1,212  (6.16%) | 1,266  (6.43%) | 1,259  (6.4%) |
