# Supplementary figures and images for: Transcriptomic Analysis Provides Novel Insights into Heat Stress Responses in Sheep
Source: Animals (Basel). 2019 Jun 24;9(6):387. doi: 10.3390/ani9060387 (PMC6617286; doi:10.3390/ani9060387)

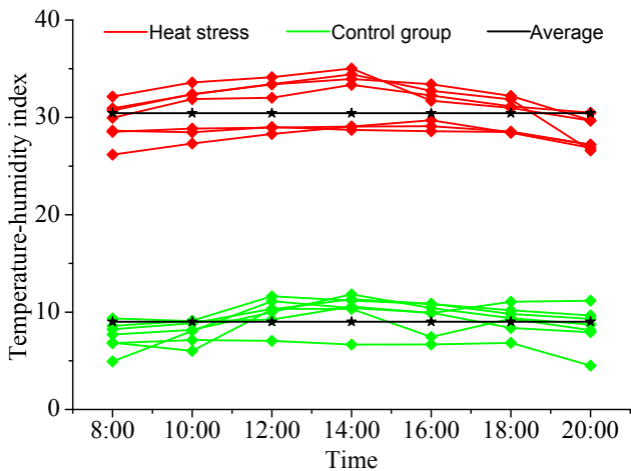

Supplement: Supplementary file 1 [file animals-09-00387-s001.zip › Supplementary/Supplementary Figure S1.pdf]
